# Effect of Dementia on the Use of Drugs for Secondary Prevention of Ischemic Heart Disease

**DOI:** 10.1155/2014/897671

**Published:** 2014-02-25

**Authors:** Nicole R. Fowler, Amber E. Barnato, Howard B. Degenholtz, Angela M. Curcio, James T. Becker, Lewis H. Kuller, Oscar L. Lopez

**Affiliations:** ^1^Division of General Internal Medicine, Department of Medicine, School of Medicine, University of Pittsburgh, Pittsburgh, PA 15213, USA; ^2^Department of Health Policy and Management, Graduate School of Public Health, University of Pittsburgh, Pittsburgh, PA 15261, USA; ^3^Center for Bioethics and Health Law, University of Pittsburgh, Pittsburgh, PA 15260, USA; ^4^College of Medicine, Drexel University, Philadelphia, PA 19129, USA; ^5^Department of Psychiatry, School of Medicine, University of Pittsburgh, Pittsburgh, PA 15213, USA; ^6^Department of Epidemiology, Graduate School of Public Health, University of Pittsburgh, Pittsburgh, PA 15261, USA; ^7^Department of Neurology, School of Medicine, University of Pittsburgh, Pittsburgh, PA 15213, USA

## Abstract

*Background*. Dementia and cardiovascular disease (CVD) are frequently comorbid. The presence of dementia may have an effect on how CVD is treated. *Objective*. To examine the effect of dementia on the use of four medications recommended for secondary prevention of ischemic heart disease (IHD): angiotensin-converting enzyme inhibitors, beta-blockers, lipid-lowering medications, and antiplatelet medications. *Design*. Retrospective analysis of data from the Cardiovascular Health Study: Cognition Study. *Setting and Subjects*. 1,087 older adults in four US states who had or developed IHD between 1989 and 1998. *Methods*. Generalized estimating equations to explore the association between dementia and the use of guideline-recommended medications for the secondary prevention of IHD. *Results*. The length of follow-up for the cohort was 8.7 years and 265 (24%) had or developed dementia during the study. Use of medications for the secondary prevention of IHD for patients with and without dementia increased during the study period. In models, subjects with dementia were not less likely to use any one particular class of medication but were less likely to use two or more classes of medications as a group (OR, 0.60; 95% CI, 0.36–0.99). *Conclusions*. Subjects with dementia used fewer guideline-recommended medications for the secondary prevention of IHD than those without dementia.

## 1. Introduction

Among medicare beneficiaries diagnosed with dementia, 30% also have a diagnosis of ischemic heart disease (IHD) [1], and as many as 54% have had a myocardial infarction at some point in their lives [2], making them candidates for the use of medications for the secondary prevention of IHD. There appears to be a link between cardiovascular disease (CVD) [3] and the development of clinical dementia, and the aggressive treatment of CVD may delay or reduce the risk of developing dementia [4–6]. For example, studies show an inverse relationship between statin use and risk for Alzheimer's disease, possibly due to the role that lipids may play in the development of this disease [7, 8].

Additionally, dementia in older adults has an effect on the management of comorbid cardiovascular disease: patients with myocardial infarction are half as likely to receive invasive cardiac procedures if they have dementia listed in their hospital chart [9]; community-dwelling patients with a diagnosis of dementia are less likely to use either cardiovascular medications [10] or a lipid-lowering medication [11], and even after controlling for IHD, use of cardiovascular medications is lower in nursing home residents with dementia, especially severe dementia [12].

While the presence of clinical dementia plays a role in decisions to use invasive procedures and medications for the management of CVD, no studies, to date, have examined and compared the use of medications recommended for the secondary prevention of IHD in a population with and without dementia over time. We used an epidemiologic cohort of subjects serially assessed for dementia and IHD to explore the relationship between dementia and the use of guideline-recommended medications for the secondary prevention of IHD between 1989-1998, a decade with substantial growth in the use of these medications. We hypothesized that use of these medications for the secondary prevention of IHD would be lower in individuals with dementia than in individuals without dementia.

## 2. Methods

### 2.1. Setting, Participants, and Study Design

We conducted a retrospective analysis of prospectively collected data from the Cognition Study within the Cardiovascular Health Study-Cognition Study (CHS-CS) [13, 14]. We used CHS-CS data concerning dementia, IHD, medication usage for the secondary prevention of IHD, and comorbidity status.

The CHS is a longitudinal, observational, community-based study of the onset, progression, and course of cardiovascular disease in 5,201 older men and women from four sites: Forsyth County, North Carolina; Sacramento, California; Washington County, Maryland; and Pittsburgh, Pennsylvania [14]. The CHS cohort was aged 65 and older at enrollment in 1989-1990 and was supplemented with 687 men and women during a minority participant recruitment effort at these same sites in 1992-1993.

Each year, the CHS-CS investigators captured data on the study participants' cognitive status by administering the Modified Mini-Mental State Examination (3MSE) [15] and the Digit Symbol Substitution Test [16]. To obtain additional information on the cognition of participants, the investigators administered the Informant Questionnaire on Cognitive Decline in the Elderly [17] and the Dementia Questionnaire [18] to proxies (e.g., spouses or children of participants). The CHS-CS protocol for the classification of dementia was previously reported [19].

The CHS investigators collected detailed diagnostic information about the study participants' cardiovascular health, including clinical, radiologic, and laboratory data. They defined prevalent cardiovascular disease as a history of myocardial infarction, angina, congestive heart failure, or stroke, as indicated by participant self-report and confirmed by a medical record review, the results of a baseline electrocardiogram, or both [20]. Incident cardiovascular disease is defined as the onset of any of these conditions after baseline assessment, subject to adjudication at the field sites with the use of information from treating physicians or from hospital discharge summaries. For this study, we defined prevalent IHD as history of myocardial infarction and angina (stable and unstable) indicated by CHS and incident IHD as the onset of either of these conditions after baseline assessment.

To ascertain information about the participants' use of prescription medications, members of the CHS team performed in-person (home or clinic) medication inventories each year [21]. In cases when the medication information could not be obtained from the participant (e.g., too cognitively impaired), a proxy provided the information for the medication inventory. The CHS-CS protocol did not include collecting data on prescriptions written by physicians. Details about the medication use assessment and validation of this method for estimating drug exposure were previously reported [21].

### 2.2. Independent Variables

For our primary analyses, the independent variable was dementia status (present/absent) of study participants with IHD for each year of longitudinal follow-up, as classified by the CHS-CS investigators. For participants with prevalent cases of dementia, we assigned their study baseline year as the year of onset. For participants with incident cases of dementia, we used the onset date determined by the CHS-CS investigators. If participants had mild cognitive impairment but did not convert to dementia during the study period, we considered them to be without dementia. Patients contributed multiple years of data with an average of 8.7 years of follow-up and could have been demented throughout or nondemented throughout or could have converted from non-demented to demented during the observation period.

As a sensitivity analysis, we explored an alternate specification of dementia, presence of dementia from the perspective of the subject or their proxy using any of the following items on the Dementia Questionnaire: a proxy report of memory or cognition problem, an indication that the participant had been told by a physician that they had memory or thinking problems, or a report that the participant had been prescribed a medication for memory. We used this variable because the CHS-CS cognitive diagnosis— a research-based assessment—may not have been known by the subject, proxy, or the community physician responsible for prescribing the medications, whereas the Dementia Questionnaire items directly measure proxy and/or physician recognition of impairment. The CHS study did not collect data on participants' treating physicians or the prescribing physician for medications.

### 2.3. Dependent Variables and Covariates

As dependent variables (main outcome measures) for our study, we used four classes of guideline-recommended cardiovascular medications: ACE inhibitors, beta-blockers, lipid-lowering medications, and antiplatelet medications. We selected these classes for two reasons: they had the strongest evidence of reducing mortality from IHD following a myocardial infarction at the time of the CHS, and they were included in clinical practice guidelines for the treatment and management of patients with IHD during all or some of the study period [22–25].

In unadjusted models, we compared medication use for two representative years: 1992 (study year 5 of original sample and baseline year for supplemented minority cohort) and 1998 (study year 11 and final study year for full sample). In our primary multivariable analyses, we determined whether participants used drugs from one or more of the four drug classes. To test for use separately and jointly, we dichotomized the variables as taking a drug from a particular class (yes/no) or taking drugs from two or more classes (yes/no) during each study year. Our models for ACE inhibitors, beta-blockers, and antiplatelet medications were from 1989 to 1998. Our model for lipid-lowering medications over time was restricted to the seven-year period from 1992 to 1998, since the use of these drugs was not standard practice until later in the study period.

Covariates tested for inclusion in each final model included age, sex, race, education level, income level, functional status (e.g., activities of daily living), clinically important factors (e.g., atrial fibrillation, congestive heart failure, stroke, depression, diabetes, hypertension, hyperlipidemia, and kidney disease), and study site.

### 2.4. Statistical Analyses

To characterize the study sample in terms of sociodemographic data and use of medications (ACE inhibitors, beta-blockers, lipid-lowering medications, antiplatelet medications, all other cardiac drugs, and total number of prescription medications), we used descriptive statistics. Chi-square tests for categorical variables and *t*-tests for continuous variables were used to compare the sociodemographic and medication data for participants with and without dementia, at different time points. Where nonparametric procedures were indicated, we used Fisher's exact tests for analysis. We examined the relationship between dementia status and use of medication for the secondary prevention of IHD by calculating the odds that individuals with dementia would be taking drugs from one or more classes of guideline-recommended medications (relative to those without dementia). To test differences in use of any drugs compared to no use, we created an “any use” variable for the four classes; we then modeled use of each drug individually and then use of any two classes together. Then, to better understand the specific role of certain covariates as confounders, we examined the unadjusted odds ratio for each covariate and the change in the ratio after adjusting for dementia status. In the final models, we included all demographic and clinical covariates that had clinical relevance or a strong association (*P* ≤ 0.10) with the use of ACE inhibitors, beta-blockers, lipid-lowering medications, or antiplatelet medications.

To model the use of the four classes of drugs individually and as a group of two or more over time, we fitted a generalized estimating equation (GEE) with binomial distribution, logit link function, and autoregressive correlation structure. For the autoregressive correlation structure, we selected a lag period of 1 year, given that the likelihood of being treated with a particular drug was higher if the same drug was used the previous year. Because GEE models assume missing data completely at random, we created weights for each observation that were inversely proportional to the probability of missing. Only participants with a determination of IHD were included in the model, so participants with incident IHD were not included until their incident year.

We used Stata/SE version 12 (StataCorp, College Station, Texas) to perform all analyses and considered a *P* value of <0.05 as significant.

## 3. Results

### 3.1. Characteristics of the Study Sample

Our study sample consisted of the 1,087 CHS-CS participants who had IHD; 591 subjects had IHD at baseline, and 496 developed IHD during the study period. The average follow-up period for the cohort was 8.7 years. The mean age of the study sample at baseline was 76.6 years. Of the 1,087 participants, 567 (52%) were male, 928 (85%) were white, 617 (57%) had a high school education or less, 786 (72%) had an annual income of $34,999 or less, and 265 (24%) had dementia identified by the CHS-CS either at baseline or later during the study (Table 1). Among the 1,087, 118 (11%) had a family member or other proxy indicated on the Dementia Questionnaire that they were aware that the participant had a cognitive impairment or that they were taking a cognitive enhancement medication (i.e., Donepezil and Memantine—1996 and later).

### 3.2. Comparison of Groups

Table 1 compares sociodemographic characteristics and functional and comorbidity status of participants without dementia and participants with dementia in two representative years, 1992 (study year 5 of original sample and baseline year for supplemented minority cohort) and 1998 (study year 11 and final study year for full sample). In each year, partcipants with dementia were significantly older, were more likely to have a high school education or less, and have lower activities of daily living score. Participants with and without dementia differed regarding comorbidity status. In both year 5 and year 11, participants with dementia were significantly more likely to have a history of atrial fibrillation, congestive heart failure, kidney disease, depression, and stroke.

Table 2 compares medication use of participants in these same years. Overall, the use of guideline-recommended medications for the secondary prevention of IHD for patients with and without dementia increased over time but was lower for those with dementia compared to those without dementia. In contrast, the use of other cardiac medications did not show substantial secular growth and they were used at similar or higher rates among those with dementia compared to those without dementia.  Table 3 shows results from the longitudinal multivariable models that adjusted for age, race, sex, hypertension, diabetes, atrial fibrillation, congestive heart failure, study site, interaction term for dementia and age, and study year. We found that participants with dementia were no more or less likely to use any one drug class, but they were significantly less likely to use two or more guideline-recommended medications (odds ratio (OR), 0.60; 95% confidence interval (CI), 0.35–0.99). Results for other covariates indicate that being nonwhite (OR, 2.26; 95% CI 1.34–3.86) and older (OR, 0.97; 95% CI 0.92–0.99) was also independently associated with less use of two or more guideline-recommended medications.

### 3.3. Effects of Proxy-Recognized Dementia

In sensitivity analyses, we found that whether or not a participant's family member or an other proxy recognized the participant's cognitive impairment had no effect on the participant's use of guideline-recommended medications (results not shown).

## 4. Discussion

To our knowledge, this is the first study to examine the effect of dementia on the use of guideline-recommended medications for the secondary prevention of IHD. During the study period, the proportion of all patients with IHD who reported using a guideline-recommended mediation increased substantially; however, those with dementia were less likely than those without dementia to use these medications. In adjusted analyses, individuals with dementia identified by the CHS-CS were less likely than individuals without dementia to use guideline-recommended drugs from two or more classes. This was not driven by lower rates of using drugs from any particular class. Cognitive impairment acknowledged by proxy on the Dementia Questionnaire did not impact use of drugs from any particular class individually or as a group.

Our study did not examine why there were differences in the use of medications between individuals with and without dementia or differences in prescribing or adherence, but several related points about our study limitations and about physicians' knowledge of dementia and prescribing practices in general should be considered.

First, in our main study analyses, the dementia status was determined by a detailed CHS-CS protocol, yet the individuals in our study were being treated by their own community-based physicians. Research shows that impaired cognitive status goes unrecognized by primary care physicians in 30%–50% of cases, [26] so it is possible that the physicians who prescribed medications for IHD were not aware of their patients' dementia status [26, 27]. We should note, however, that in 1997-1998 (the same year the Dementia Questionnaire was administered to proxies) the CHS-CS investigators informed the participants' physicians if test results showed strong evidence of cognitive impairment.

Second, we were unable to measure who the prescribing physician(s) was for each of these medications and the reasons why a patient may have reported less use of a guideline-recommended medication (e.g., lack of an indication, never received a prescription, nonadherence, patient preferences, and poor prognosis) [28]. Therefore, we could not make any conclusions if the lower use was appropriate or inappropriate. Because the medications recommended for the secondary prevention of IHD are relatively low-cost and likely considered to be low-burden and low-risk, we might expect them to be considered for any patient with IHD. Yet, dementia is known to reduce life expectancy, and the progressive nature of this condition may reduce the priority placed on secondary prevention of IHD. It is possible that this reprioritization of care would include evidence-based management for comorbidities and the changing of treatment plans during the course of dementia to match patient life expectancy, preferences, and treatment goals.

Third, medications for the secondary prevention of IHD are generally recommended to continue indefinitely. Their use may be challenging for any patient who is already receiving multiple drugs or if a patient had been taken off of a drug due to side effects. It can be particularly challenging for patients with dementia or their caregivers to have prescriptions filled and to take medications as instructed.

Fourth, although physicians are more reluctant to stop prescribing a medication than they are to not prescribe it in the first place, guideline-recommended medications could ethically be withdrawn if necessary (e.g., if the patient were using the drug erratically) [29]. Nevertheless, some physicians may not feel comfortable stopping medications, either because of the risk of adverse events or because they believe that stopping the drugs might be seen as a sign of “giving up” or contradicting earlier advice. In a follow-up study using the same dataset, we will investigate the relationship of the timing of onset of both dementia and IHD to measure if those who develop IHD prior to dementia are more likely to use these medications compared to those who develop IHD after dementia.

Fifth, the data used for this study are from 1989 to 1998, and use of these medications in current practice is higher. Also, we did not include use of angiotensin receptor blockers which have been shown in recent studies to reduce the risk of dementia [30]. Nonetheless, the objective of this study was to determine differences in use between those with and without dementia, not rates of use per se. Although the data used in these analyses are from 1989 to 1998, we believe our findings are relevant given the evidence regarding the aggressive treatment of CVD and a possible delay or reduction in the risk of developing dementia. Additionally, we believe our findings are valid because differences in the treatment and management of comorbidities among older adults with dementia persist and continue to be reported in the literature [31, 32] despite greater overall health care utilization for older adults with dementia. It would be important for future research to assess the magnitude and consequences of these disparities, as well as the source, and whether differences are a reflection of differences in patient goals of care or possible provider biases.

In summary, the dementia status of patients is associated with less use of drugs for secondary prevention of IHD. Further studies are needed to determine why this is true for these particular drugs and whether it is true for other prescription drugs. Additionally, future studies investigating prescription patterns and subsequent patient adherence as a function of cognitive status could inform sources of the disparity. This line of inquiry could prove valuable for heightening physicians' awareness of their patients' cognitive status and its role in the overall management of their health. Our study also provides preliminary information for efforts to develop interventions to improve how physicians present information about treatment options and outcomes to patients with dementia and other comorbidities. Based on our findings, we believe it is important that future studies investigate sources of variation in the use of medications and the possible factors that influence medical decision making for patients with dementia.

## Figures and Tables

**Table 1:** Characteristics of study participants in 1992 and 1998, stratified by dementia status*.

Characteristic	1992 (study year 5)^†^ Number (%)			1998 (study year 11)^‡^ Number (%)		
	Without dementia (*n* = 1,005)	With dementia (*n* = 82)	*P* value	Without dementia (*n* = 672)	With dementia (*n* = 190)	*P* value
Age, years			<0.0001			<0.0001
≤75	562 (55.9)	25 (31.0)		76 (11.3)	7 (3.7)	
76–85	408 (40.6)	44 (54.3)		447 (66.6)	89 (47.0)	
86–95	26 (2.6)	12 (14.8)		92 (13.7)	59 (31.1)	
≥96	9 (1.0)	0 (0)		56 (8.4)	35 (18.4)	
Sex			0.10			0.46
Male	517 (51.4)	50 (61.0)		337 (50.1)	101 (53.2)	
Female	488 (48.6)	32 (39.0)		335 (49.9)	89 (46.8)	
Race			<0.0001			0.0002
White	873 (87.9)	55 (67.1)		582 (86.6)	143 (75.3)	
Nonwhite	132 (13.1)	27 (32.9)		90 (13.4)	47 (24.7)	
Education level			0.006			0.004
≤High school	559 (55.7)	58 (71.6)		358 (53.4)	124 (65.3)	
>High school	444 (44.3)	23 (28.4)		313 (46.7)	66 (34.7)	
Annual income			0.49			0.02
≤$34,999	724 (72.0)	62 (75.6)		473 (70.4)	150 (79.0)	
>$35,000	281 (28.0)	20 (24.4)		199 (29.6)	40 (21.0)	
ADL score, mean (SD)	0.17 (0.6)	0.54 (1.1)	<0.0001	0.41 (0.9)	1.63 (2.0)	<0.0001
COPD	167 (16.8)	10 (12.5)	0.31	150 (22.3)	35 (18.4)	0.28
Depression^§^	270 (27.3)	40 (50.6)	<0.0001	166 (29.4)	52 (51.5)	<0.0001
Diabetes	167 (16.6)	15 (18.3)	0.70	161 (24.0)	44 (23.3)	0.83
Hyperlipidemia	592 (59.2)	54 (67.5)	0.15	408 (61)	124 (66.3)	0.18
Hypertension	470 (47.5)	41 (51.2)	0.51	336 (60.0)	72 (62.1)	0.68
Kidney disease	183 (18.2)	26 (31.7)	0.003	90 (13.4)	43 (22.6)	0.002
Stroke	79 (7.8)	24 (29.3)	<0.0001	138 (21.3)	60 (33.9)	<0.001
Comorbidity			0.002			0.02
<3 comorbid conditions	968 (96.3)	73 (89)		598 (89.0)	157 (82.6)	
≥3 comorbid conditions	37 (3.68)	9 (11)		74 (11.0)	33 (17.4)	
Died	0 (0)	1 (1.2)	0.08	28 (4.2)	29 (59.3)	<0.0001
Follow-up, years, mean (SD)	5.20 (1.81)	0.59 (0.06)	<0.0001	6.27 (0.63)	3.14 (1.34)	<0.0001

ADL: activities of daily living; CES-D: Center for Epidemiological Studies Depression Scale; COPD: chronic obstructive pulmonary disease; SD: standard deviation.

*Because of rounding, percentages may not total 100.

^†^Study year 5 for initial sample and baseline year for supplemented minority cohort.

^‡^Final study year for full sample.

^§^Depression indicated with a score of >7 on the CES-D.

**Table 2:** Unadjusted use of guideline-recommended medications by study participants in 1992 and 1998, stratified by dementia status.

Medication	1992 (study year 5)* Number (%)			1998 (study year 11)^†^ Number (%)		
	Without dementia (*n* = 1,005)	With dementia (*n* = 82)	*P* value	Without dementia (*n* = 672)	With dementia (*n* = 190)	*P* value
Medications for IHD						
ACE inhibitor	131 (13.2)	15 (18.3)	0.20	146 (24.4)	35 (25.0)	0.89
Beta-blocker	221 (22.3)	8 (9.8)	0.008	220 (36.8)	36 (25.7)	0.01
Lipid-lowering medication^‡^	102 (10.3)	3 (3.7)	0.05	184 (30.8)	22 (15.7)	<0.001
Antiplatelet medication^§^	511 (51.5)	37 (45.1)	0.27	360 (63.8)	69 (58.0)	0.23
Use of two or more medications for IHD	266 (26.8)	15 (18.3)	0.09	300 (50.1)	50 (35.5)	0.002
Use of other cardiac medications						
Calcium channel blocker	348 (35.1)	27 (32.9)	0.70	205 (34.3)	43 (30.7)	0.42
Diuretic	297 (30.0)	31 (37.8)	0.14	217 (36.3)	52 (37.1)	0.85
Digoxin	149 (15.0)	18 (22.0)	0.10	116 (19.4)	30 (21.4)	0.59
Nitrate	250 (25.2)	18 (22.0)	0.52	158 (26.4)	44 (31.4)	0.23
Class 1A antiarrhythmic agent	36 (3.6)	2 (2.4)	0.76	2 (0.33)	4 (2.9)	0.01
Total number of all prescription medications, mean (SD)	0.99 (0.9)	0.79 (0.7)	0.04	1.57 (1.0)	1.27 (1.0)	0.003

ACE: angiotensin-converting enzyme; IHD: ischemic heart disease; SD: standard deviation.

*Study year 5 for initial sample and baseline year for supplemented minority cohort.

^†^Final study year for full sample.

^‡^Lipid-lowering medications included statins (3-hydroxy-3-methyglutaryl-coenzyme A [HMG-CoA] reductase inhibitors) and nonstatins.

^§^Antiplatelet medications included cyclooxygenase inhibitors (e.g., aspirin) and adenosine diphosphate (ADP) receptor inhibitors.

**Table 3:** Association between dementia and use of one or more classes of guideline-recommended medications for secondary prevention of ischemic heart disease by study participants, 1989 to 1998.

Variable	Odds ratio (95% confidence interval)				
	Use of an ACE inhibitor	Use of a beta-blocker	Use of a lipid-lowering medication*	Use of an antiplatelet medication^†^	Use of two or more classes of guideline-recommended medications
Dementia	1.19 (0.79–1.77)	0.97 (0.65–1.44)	0.76 (0.37–1.55)	0.74 (0.45–1.23)	0.60 (0.35–0.99)^‡^
Age	0.99 (0.94–1.03)	0.98 (0.94–1.02)	0.99 (0.93–1.05)	0.97 (0.94–0.99)^‡^	0.97 (0.92–0.99)^‡^
Age and dementia interaction	1.01 (0.92–1.10)	1.03 (0.98–1.07)	0.99 (0.92–1.07)	1.06 (0.99–1.14)	1.10 (1.03–1.19)^‡^
White race	1.93 (0.95–3.91)	0.91 (0.43–1.92)	2.28 (0.99–5.20)^‡^	2.66 (1.65–4.30)^‡^	2.26 (1.34–3.86)^‡^
Male sex	1.12 (0.69–1.82)	1.32 (0.78–2.23)	1.21 (0.58–2.54)	1.40 (1.00–1.94)	1.24 (0.80–1.90)
Atrial fibrillation	1.16 (0.71–1.90)	0.95 (0.53–1.70)	0.29 (0.15–0.56)^‡^	0.98 (0.70–1.38)	1.03 (0.66–1.61)
CHF	2.87 (1.33–6.55)^ ‡^	0.40 (0.11–1.28)	1.42 (0.37–5.50)	0.95 (0.49–1.84)	1.12 (0.50–2.53)
Hypertension	1.12 (1.00–1.23)	1.15 (1.08–1.24)^‡^	1.47 (1.00–2.15)^‡^	1.07 (0.96–1.19)	1.16 (1.05–1.30)^‡^
Diabetes	1.21 (1.00–1.46)^ ‡^	0.86 (0.78–0.96)^‡^	1.14 (0.96–1.37)	1.06 (0.91–1.23)	1.08 (0.94–1.23)

ACE: angiotensin-converting enzyme; CHF: congestive heart failure.

*Lipid-lowering medications included statins (3-hydroxy-3-methyglutaryl-coenzyme A [HMG-CoA] reductase inhibitors) and nonstatins. The model for these medications was restricted to data from 1992 to 1998.

^†^Antiplatelet medications included cyclooxygenase inhibitors (e.g., aspirin) and adenosine diphosphate (ADP) receptor inhibitors.

^‡^ *P* < 0.05.

## References

[1] Maslow K (2004). Dementia and serious coexisting medical conditions: a double whammy. *Nursing Clinics of North America*.

[2] Brauner DJ, Muir JC, Sachs GA (2000). Treating nondementia illnesses in patients with dementia. *The Journal of the American Medical Association*.

[3] Tilvis RS, Kähönen-Väre MH, Jolkkonen J, Valvanne J, Pitkala KH, Strandberg TE (2004). Predictors of cognitive decline and mortality of aged people over a 10-year period. *Journals of Gerontology A*.

[4] Bunch TJ, Weiss JP, Crandall BG (2010). Atrial fibrillation is independently associated with senile, vascular, and Alzheimer’s dementia. *Heart Rhythm*.

[5] Hofman A, Ott A, Breteler MMB (1997). Atherosclerosis, apolipoprotein E, and prevalence of dementia and Alzheimer’s disease in the Rotterdam Study. *The Lancet*.

[6] Rosenberg PB, Mielke MM, Tschanz J (2008). Effects of cardiovascular medications on rate of functional decline in alzheimer disease. *American Journal of Geriatric Psychiatry*.

[7] Jick H, Zornberg GL, Jick SS, Seshadri S, Drachman DA (2000). Statins and the risk of dementia. *The Lancet*.

[8] Zamrini E, McGwin G, Roseman JM (2004). Association between statin use and Alzheimer’s disease. *Neuroepidemiology*.

[9] Sloan FA, Trogdon JG, Curtis LH, Schulman KA (2004). The effect of dementia on outcomes and process of care for medicare beneficiaries admitted with acute myocardial infarction. *Journal of the American Geriatrics Society*.

[10] Schmader KE, Hanlon JT, Fillenbaum GG, Huber M, Pieper C, Horner R (1998). Medication use patterns among demented, cognitively impaired and cognitively intact community-dwelling elderly people. *Age and Ageing*.

[11] Rodriguez EG, Dodge HH, Birzescu MA (2002). Use of lipid-lowering drugs in older adults with and without dementia: a community-based epidemiological study. *Journal of the American Geriatrics Society*.

[12] Landi F, Gambassi G, Lapane KL (1998). Comorbidity and drug use in cognitively impaired elderly living in long-term care. *Dementia and Geriatric Cognitive Disorders*.

[13] Fried LP, Borhani NO, Enright P (1991). The cardiovascular Health Study: design and rationale. *Annals of Epidemiology*.

[14] Lopez OL, Kuller L, Fitzpatrick AL, Ives D, Becker JT, Beauchamp N (2003). Evaluation of dementia in the Cardiovascular Health Cognition Study. *Neuroepidemiology*.

[15] Folstein MF, Folstein SE, McHugh PR (1975). Mini-mental state: a practical method for grading the cognitive state of patients for the clinician. *Journal of Psychiatric Research *.

[16] Wechsler D (1981). *WAIS-R Manual*.

[17] Jorm AF, Jacomb PA (1989). The informant questionnaire on cognitive decline in the elderly (IQCODE): socio-demographic correlates, reliability, validity and some norms. *Psychological Medicine*.

[18] Kawas C, Segal J, Stewart WF, Corrada M, Thal LJ (1994). A validation study of the dementia questionnaire. *Archives of Neurology*.

[19] Fitzpatrick AL, Kuller LH, Ives DG (2004). Incidence and prevalence of dementia in the Cardiovascular Health Study. *Journal of the American Geriatrics Society*.

[20] Psaty BM, Kuller LH, Bild D (1995). Methods of assessing prevalent cardiovascular disease in the cardiovascular health study. *Annals of Epidemiology*.

[21] Psaty BM, Lee M, Savage PJ (1992). Assessing the use of medications in the elderly: methods and initial experience in the cardiovascular health study. *Journal of Clinical Epidemiology*.

[22] Pollack CV, Gibler WB (2001). 2000 ACC/AHA guidelines for the management of patients with unstable angina and non-ST-segment elevation myocardial infarction: a practical summary for emergency physicians. *Annals of Emergency Medicine*.

[23] Smith S.C. J, Blair SN, Bonow RO (2001). AHA/ACC guidelines for preventing heart attack and death in patients with atherosclerotic cardiovascular disease: 2001 update: a statement for healthcare professionals from the american heart association and the american college of cardiology. *Circulation*.

[24] Ryan TJ, Anderson JL, Antman EM (1996). ACC/AHA guidelines for the management of patients with acute myocardial infarction. A report of the American College of Cardiology/American Heart Association Task Force on Practice Guidelines (Committee on Management of Acute Myocardial Infarction). *Journal of the American College of Cardiology*.

[25] Hunt SA, Abraham WT, Chin MH (2005). ACC/AHA guidelines for the diagnosis and management of chronic heart failure in the adult: a report of the American College of Cardiology/American Heart Association Task Force on Practice Guidelines (Committee on the Evaluation and Management of Heart Failure). *Journal of the American College of Cardiology*.

[26] Boustani M, Peterson B, Hanson L, Harris R, Lohr KN (2003). Screening for dementia in primary care: a summary of the evidence for the U.S. preventive services task force. *Annals of Internal Medicine*.

[27] Callahan CM, Hendrie HC, Tierney WM (1995). Documentation and evaluation of cognitive impairment in elderly primary care patients. *Annals of Internal Medicine*.

[28] Campbell N, Boustani M, Skopelja EN (2012). Medication adherence in older adults with cognitive impairment: a systematic evidence-based review. *American Journal of Geriatric Pharmacotherapy*.

[29] Rhymes JA, McCullough LB, Luchi RJ, Teasdale TA, Wilson N (2000). Withdrawing very low-burden interventions in chronically ill patients. *The Journal of the American Medical Association*.

[30] Li N, Lee A, Whitmer RA (2010). Use of angiotensin receptor blockers and risk of dementia in a predominantly male population: prospective cohort analysis. *British Medical Journal*.

[31] Thorpe CT, Thorpe JM, Kind AJ, Bartels CM, Everett CM, Smith MA (2012). Receipt of monitoring of diabetes mellitus in older adults with comorbid dementia. *Journal of the American Geriatrics Society*.

[32] Morrison RA, Siu LA (2000). A comparison of pain and its treatment in advanced dementia and cognitively intact patients with hip fracture. *Journal of Pain and Symptom Management*.

